# Assessment of postoperative complications using E-PASS and APACHE II in patients undergoing oral and maxillofacial surgery

**DOI:** 10.1186/s13037-018-0152-6

**Published:** 2018-04-05

**Authors:** Kiyohide Ishihata, Yasuyuki Kakihana, Takuya Yoshimura, Juri Murakami, Soichiro Toyodome, Hiroshi Hijioka, Etsuro Nozoe, Norifumi Nakamura

**Affiliations:** 1 Department of Oral and Maxillofacial Surgery, Field of Maxillofacial Rehabilitation, Kagoshima University Graduate School of Medical and Dental Sciences, 8-35-1 Sakuragaoka, Kagoshima, 890-8544 Japan; 2 Department of Emergency and Intensive Care Medicine, Faculty of Medicine, Kagoshima University, Kagoshima, Japan

**Keywords:** Oral and maxillofacial surgery, Estimation of Physiologic Ability and Surgical Stress (E-PASS), Acute Physiology, Age, and Chronic Health Evaluation (APACHE) II, Postoperative complications

## Abstract

**Background:**

The prediction of postoperative complications is important for oral and maxillofacial surgeons. We herein aimed to evaluate the efficacy of the Estimation of Physiologic Ability and Surgical Stress (E-PASS) and Acute Physiology, Age, and Chronic Health Evaluation (APACHE) II scoring systems to predict postoperative complications in patients undergoing oral and maxillofacial surgery.

**Methods:**

Thirty patients (22 males, 8 females; mean age: 65.1 ± 12.9 years) who underwent major oral surgeries and stayed in the intensive care unit for postoperative management were enrolled in this study. Postoperative complications were discriminated according to the necessity of the therapeutic intervention by the Medical Department, i.e. according to the Clavien–Dingo classification. E-PASS and APACHE II scores as well as laboratory test values were compared between patients with/without postoperative complications.

**Results:**

Postoperative complications were developed in seven patients. The comprehensive risk score (CRS: 1.13 ± 0.24) and APACHE II score (13.0 ± 2.58) were significantly higher in patients with postoperative complications than in those without ones (*p* < 0.01, *p* < 0.05, respectively). The CRS showed an appropriate discriminatory power for predicting postoperative complications (area under the curve: 0.814). Furthermore, a correlation was detected between APACHE II scores and postoperative data until C-reactive protein levels decreased to < 1.0 mg/L (*r* = 0.43, *p* < 0.05).

**Conclusion:**

The E-PASS and APACHE II scoring systems were both shown to be useful to predict postoperative complications after oral and maxillofacial surgery.

## Background

Risk management for postoperative complications is important for patients and surgeons. Surgical interventions lead to the production of proinflammatory cytokines, which may result in the development of systemic inflammatory response syndrome (SIRS) [1, 2]. These biological responses are regarded as beneficial because they increase immune functions and promote tissue repair; however, if the reserve competence of a patient cannot withstand surgical stress, homeostasis may collapse, and as a consequence, various postoperative complications may develop. Postoperative complications such as surgical site infection (SSI), aspiration pneumonia, and swallowing and breathing difficulties have a significant impact on the prognosis of patients, and as a result, increase health care costs and hospitalization [3]. Therefore, many surgeons have examined patients based on their performance statuses, biological ages, and clinical test results.

Several scoring systems have recently been developed to predict postoperative morbidity and mortality in an attempt to prevent unfavorable outcomes after general surgery; however, there have been few studies on the predictors of postoperative complications in the field of oral and maxillofacial surgery for oral surgeons. The estimation of physiologic ability and stress (E-PASS) scoring system is a useful and simple strategy to predict postoperative mortality and morbidity [2, 4]. It evaluates the physiological condition of a patient and surgical invasion and precisely reflects the general condition of a patient in a perioperative setting. Acute Physiology, Age, and Chronic Health Evaluation (APACHE) II, a severity of disease classification system that use basic physiological principles, has frequently been applied in many Intensive Care Units (ICU) to stratify prognosis of acute ill patients [5, 6].

Since the oral and maxillofacial regions with its important arteries and veins or nerves play a major role in ingestion and breathing, reliable predictors for postoperative complications are needed. However, few studies have been performed to identify predictive risk factors related to postoperative complications in patients who undergo oral and maxillofacial surgery.

Thus, we herein aimed to identify risk factors that correlate with postoperative complications in patients who underwent oral and maxillofacial surgery with relatively high surgical stress in our hospital. Furthermore, this is the first attempt to assess the utility of the E-PASS and APACHE II scoring systems in this field.

## Methods

### Patient characteristics

Thirty patients who were treated for oral cancer or oral benign tumors with resection and reconstruction in the Department of Oral and Maxillofacial Surgery at a university hospital in Kagoshima, and admitted to the ICU for postoperative management between 2013 and 2015 were reviewed retrospectively. An Institutional Review Board approved this retrospective observational study. Patients included 22 men and eight women with a mean age of 66.9 ± 11.0 years. The original disease was a malignant tumor in 29 patients and benign tumor in one patient. Radical neck dissection was performed on 21 patients, supraomohyoid neck dissection on six patients, and upper neck dissection on one patient. Regarding reconstruction, a pectoralis major myocutaneous flap was used in nine patients, forearm flap in 9, latissimus dorsi flap in 6, and delto-pectoral flap in three patients. In all patients, the same operator team at the same hospital performed tumor resection, neck resection, and flap reconstruction. Duration of follow-up period was 2.5 years.

Postoperative complications were discriminated according to the necessity of the therapeutic intervention by the Medical Department, i.e. according to the Clavien – Dingo classification [7]. In this classification, surgical complications were categorized from grade 1 to 5 based on the invasiveness of the treatment required. Grade 1 requires no treatment; grade 2 needs medical therapy; grade 3a requires surgical, endoscopic, or radiological intervention, but no general anesthesia; grade 3b requires general anesthesia; grade 4 represents life-threatening complications that require intensive care; and grade 5 represents complications leading to patient death. In this study, the grade 2 or above patients were determined to have postoperative complications. Thus, patients were classified into two groups based on whether they developed postoperative complications.

### Scoring systems and laboratory test values

#### E-PASS and APACHE II scoring systems

We investigated E-PASS and APACHE II score variables and evaluated postoperative courses. The development of E-PASS has already been described in detail [2]. Briefly, the original E-PASS consisted preoperative risk score (PRS), which reflects reserve capacity, surgical stress score (SSS), which reflects surgical stress, and the comprehensive risk score (CRS), in which PRS and SSS are combined. APACHE II uses a point score based on 12 routine physiological measurements (ranges from 0 to 60), age score (from 0 to 6), and previous health status score (from 0 to 5) to provide a general measure of the severity of disease [5] (Table 1).

**Table 1:** Estimation of Physiologic Ability and Surgical Stress (E-PASS) and Acute Physiology and Chronic Health Evaluation (APACHE) II scoring systems

The E-PASS score consists of three parts for estimation of physiologic ability (PRS), surgical stress (SSS), and their comprehensive score(CRS). The formula for each score was as follows:									
PRS = − 0.0686 + 0.00345X1 + 0.323X2 + 0.205X3 + 0.153X4 + 0.148X5 + 0.0666X6									
XI: age									
X2: absence (0) or presence (1) of severe heart disease									
X3: absence (0) or presence (I) of severe pulmonary disease									
X4: absence (0) or presence (I) of diabetes mellitus									
X5: performance status index (0–4)									
X6: American Society of Anesthesiologists physiological status classification (1–5)									
SSS = − 0.342 + 0.0139X1 + 0.0392X2 + 0.352X3									
XI: blood loss/body weight (g/kg)									
X2: Operative time (hours)									
X3: Extent of the skin incision (0: minor incision, 1: laparotomy or thoracotomy alone, 2: both laparotomy and thoracotomy)									
CRS = − 0.328 + 0.396(PRS) + 0.976(SSS)									
The APACHE II score is the sum of the acute physiology score (vital signs, oxygenation, laboratory values), the Glasgow coma score, age, and Choronic health points. The worst values during the first 24 h in the ICU should be used. Glasgow coma score(GCS) = eye-openig score + veabal score (intubated or nonintubated) score + motor score. For CCS component of acute physiology score, subtract GCS from 15 to obtain points assigned.									
Acute Physiology Score									
Score	4	3	2	1	0	1	2	3	4
Rectal temperature (°C)	≥41	39.0~ 40.9		38.5~ 38.9	36.0~ 38.4	34.0~ 35.9	32.0~ 33.9	30.0~ 31.9	≤29.9
Mean blood pressure (mmHg)	≥160	130~ 159	110~ 129		70~ 109		50~ 69		≤49
Heart rate (beat/min)	≥180	140~ 179	110~ 139				55~ 69	40~ 54	≤39
Respiratory rate (breaths/min)	≥50	35~ 49		25~ 34	12~ 24	10~ 11	6~ 9		≤5
Arterial pH	≥7.70	7.60~ 7.69		7.50~ 7.59	7.33~ 7.49		7.25~ 7.32	7.15~ 7.24	< 7.15
Oxygenation: A-aD02 orPa02 (mmHg)									
a. FiO2 > 0.5 record A-aD02	≥500	350~ 499	200~ 349		< 200				
b. Fi02 ≤ 0.5 record Pa02					> 70	61~ 70		55~ 60	< 55
Serum sodium (mmol/L)	≥180	160~ 179	155~ 159	150~ 154	130~ 149		120~ 129	111~ 119	≤110
Serum potassium(mmol/L)	≥7.0	6.0~ 6.9		5.5~ 5.9	3.5~ 5.4	3.0~ 3.4	2.5~ 2.9		< 2.5
Serum creatinine) mg/dl)	≥3.5	2.0~ 3.4	1.5~ 1.9		0.6~ 1.4		< 0.6		
Hematocrit (%)	≥60		50~ 59.9	46~ 49.9	30~ 45.9		20~ 29.9		< 20
White blood cell count (× 1000)	≥40		20~ 39.9	15~ 19.9	3~ 14.9		1~ 2.9		< 1
Glasgow Coma Score									
Eye Opening	verbal (Non-intubated)			veabal (intubated)			Motor Activity		
4-Spontaneous	5-Oriented and talks			5-Seemsable to talk			6-Verbal command		
3-Verbal stimuli	4-Disoricnted and talks			3-Questionableabilhy to talk			5-Localizedto pain		
2-Painful stimuli	3-Inappropriate words			1-Generally unresponsive			4-Withdraws from pain		
1-No response	2-Incomprehensihle sounds						3-Decorticate		
	1-No response						2-Decerebrate		
							1-No response		
Points Assigned to Age and Chronic Disease									
Age, Years	Score								
< 45	0								
45~ 54	2								
55~ 64	3								
65~ 74	5								
≥75	6								
Chronic Health (History of Chronic Conditions)							Score		
None							0		
if the patient is admitted after elective surgery							2		
if the patient is admitted after emergency surgery or for a reason other than after elective surgery							5		

Abbreviations: *A-aD02* alveolar-arterial oxygen difference, *Fi02* fraction of inspired oxygen, *Pa02* partial pressure of oxygen

#### Laboratory test values

On the preoperative day, a physical examination of cardiac and respiratory functions was performed, and a number of parameters, such as ejection fraction (EF), vital capacity (VC), 1 sec forced expiratory volume (FEV1.0), hemoglobin (Hg), C-reactive protein (CRP) levels were measured. We also calculated the prognostic nutritional index (PNI) and body mass index (BMI) and measured prealbumin (PreAlb), transferrin (Tf), and retinol binding protein (RBP) levels as well as CRP levels from postoperative days 1–30. The PNI is calculated using the following formula: 10 × serum albumin (g/dL) + 0.005 × total lymphocyte count (per mm^3^).

#### Statistical analysis

Statistical analyses were performed using JMP version 12 software (SAS Institute Inc., Cary, NC, USA). The significance of differences between values in two different groups was assessed by the Mann - Whitney *U-*test. In a univariate analysis, a comparison of categorical variables was performed using the chi-squared test. Continuous data are expressed as the mean ± standard deviation (SD). A receiver operating characteristic (ROC) curve was created to assess the ability of E-PASS and APACHE II scores to predict the incidence of complications. A ROC curve was generated and sensitivity was plotted against specificity. The area under the ROC curve (AUC) was used to evaluate discriminatory ability to detect postoperative complications. A cut-off value corresponding to maximum sensitivity and specificity was obtained using Youden’s index from the ROC curve. The relationships between different continuous variables were quantified by Pearson’s correlation by rank. A *p*-value of less than 0.05 was considered significant.

## Results

Postoperative complications were developed in seven out of 30 patients, who were subsequently categorized into the complication group (Table 2). Among these patients, one had methicillin-resistant *Staphylococcus aureus* (MRSA) pneumonia, five had postoperative pulmonary disease, and 1 had MRSA bacteremia; there was no postoperative deaths. No significant differences were observed in age (70.2 ± 10.1 years versus 63.5 ± 13.5 years, *p* = 0.123), gender (*p* = 0.896), smoking (*p* = 0.746) or BMI (21.3 ± 3.8 kg/m^2^ versus 21.6 ± 2.6 kg/m^2^, *p* = 0.341). Among the preoperative cardiac and respiratory parameters measured, no significant differences were observed in EF (62.8 ± 15.5% versus 69.5 ± 5.5%, *p* = 0.149), VC (2.9 ± 0.9 L versus 3.2 ± 0.8 L, *p* = 0.193), FEV1.0 (2.0 ± 0.6 L versus 2.3 ± 0.7 L, *p* = 0.150), Hg (13.0 ± 2.3 g/dL versus 12.9 ± 1.7 g/dL, *p* = 0.404), or CRP (0.6 ± 1.0 mg/dL versus 0.4 ± 0.7 g/dL, *p* = 0.414) between patients with/without postoperative complications. Similarly, for the preoperative nutrition status, no significant differences were noted in PNI (46.6 ± 5.8 versus 47.3 ± 6.7, *p* = 0.5), PreAlb (22.1 ± 3.2 mg/dL versus 22.5 ± 4.6 mg/dL, *p* = 0.435), Tf (253.7 ± 54.7 mg/dL versus 226.6 ± 27.7 mg/dL, *p* = 0.136), or RBP (2.85 ± 0.5 mg/dL versus 2.90 ± 0.9 mg/dL, *p* = 0.318) between patients with/without postoperative complications. Regarding intraoperative results, the operative time and amount of blood lost during surgery, or length of hospital stay were similar between the two groups (operative time; 14.2 ± 4.7 h versus 13.5 ± 2.7 h, *p* = 0.490, blood loss; 476.7 ± 336 mg versus 405.7 ± 242 mg/dL, *p* = 0.323, length of hospital days; 65.0 ± 36.9 days versus 78.6 ± 46.2 days, *p* = 0.221) (Table 2).

**Table 2:** Preoperative laboratory data and operative findings between patients with/without postoperative complications

	Complications (+) (*n* = 7)	Complications (−) (*n* = 23)	*p*-value
Age	70.2 ± 10.1	63.5 ± 13.5	0.123
Gender			0.896
Men	5	17	
Women	2	6	
Smoking	1	4	0.746
BMI	21.3 ± 3.8	21.6 ± 2.6	0.341
EF (%)	62.8 ± 15.5	69.5 ± 5.5	0.149
VC (L)	2.9 ± 0.9	3.2 ± 0.8	0.193
%VC	97.2 ± 14.6	98.8 ± 26.0	0.206
FEV1.0 (L)	2.0 ± 0.6	2.3 ± 0.7	0.150
FEV1.0%	72.5 ± 6.7	75.9 ± 6.4	0.156
Hg (g/dL)	13.0 ± 2.3	12.9 ± 1.7	0.404
CRP (mg/dL)	0.6 ± 1.0	0.4 ± 0.7	0.414
PNI	46.6 ± 5.8	47.3 ± 6.7	0.500
PreAlb (mg/dL)	22.1 ± 3.2	22.5 ± 4.6	0.435
Tf (mg/dL)	253.7 ± 54.7	226.6 ± 27.7	0.136
RBP (mg/dL)	2.85 ± 0.5	2.90 ± 0.9	0.318
Diagnosis			
Malignant tumor	7	22	
Benign tumor	0	1	
Operative approach			
Radical neck dissection	6	15	
Supraomohyoid Neck Dissection	1	5	
Upper Neck Dissection		1	
Reconstruction method			
PMMC flap	3	6	
Forearm flap	3	6	
Latissimus dorsi flap		6	
DP flap		3	
Operative time (hr)	14.2 ± 4.7	13.5 ± 2.7	0.490
Blood loss (mg)	476.7 ± 336.6	405.7 ± 242.4	0.323
Postoperative complications			
Pneumonia	6		
MRSA bacteremia	1		
Length of hospital stay (days)	65.0 ± 36.9	78.6 ± 46.2	0.221

*BMI* body mass index, *EF* ejection fraction, *VC* vital capacity, *FEV1.0* One second forced expiratory volume, *PNI* prognostic nutritional index, *PreAlb* Prealbumin, *Tf* transferrin, *RBP* retinol binding protein, *PMMC* pectoralis major myocutaneous, *DP* Delto-pectoral, *MRSA* methicillin-resistance *Staphylococcus aureus*

**Fig. 1:**
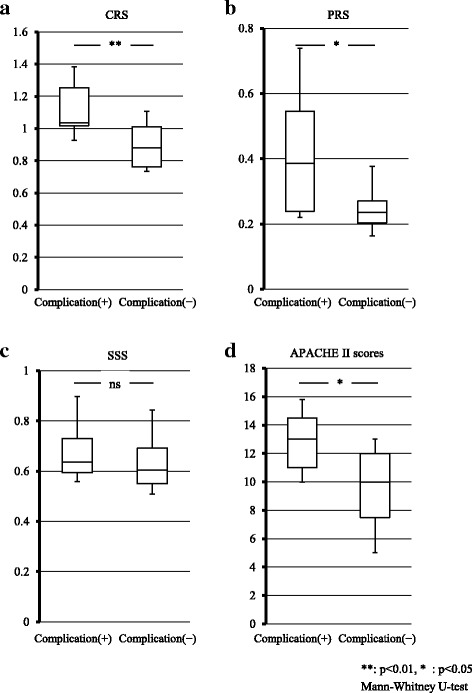
Comparison of E-PASS and APACHE II scores between patients with and without postoperative complications. Comparison of the comprehensive risk score (CRS): **a**, preoperative risk score (PRS): **b**, surgical stress score (SSS): **c** and acute physiology and chronic health evaluation (APACHE) II score: **d** between patients with and without postoperative complications. CRS (*p* < 0.01), PRS (*p* < 0.05) and APACHE II (*p* < 0.05) scores were significantly higher in patients with than in those without postoperative complications. No significant difference was observed in SSS scores (*p* = 0.20) between the 2 groups

The CRS, PRS, and APACHE II scores were significantly higher in patients with postoperative complications than in those without ones (CRS; 1.13 ± 0.24 versus 0.89 ± 0.15, *p* < 0.01, PRS; 0.45 ± 0.26 versus 0.26 ± 0.11, *p* < 0.05, APACHE II; 13 ± 2.58 versus 9.39 ± 3.43, *p* < 0.05), whereas SSS was similar between the two groups (0.69 ± 0.17 versus 0.64 ± 0.13, *p* = 0.199) (Fig. 1).

**Fig. 2:**
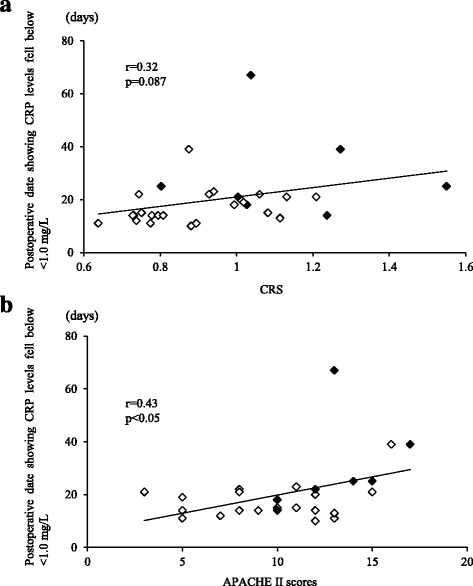
Relationship between the number of postoperative days until C-reactive protein levels decreased to < 1.0 mg/L and the comprehensive risk score (CRS) (**a**) or acute physiology and chronic health evaluation (APACHE) II score: (**b**). Correlations were observed between APACHE II and the number of postoperative days until CRP levels decreased to < 1.0 mg/L (*r* = 0.43, *p* < 0.05). No correlations were found between CRS scores and the number of postoperative days until CRP levels decreased to < 1.0 mg/L (*p* = 0.087). ◇: Without postoperative complications. ◆: With postoperative complications

The number of postoperative days until CRP levels decreased to < 1.0 mg/L correlated with APACHE II scores (*r* = 0.43, *p* < 0.05), but not the CRS (*r* = 0.32, *p* = 0.087) (Fig. 2).

**Fig. 3:**
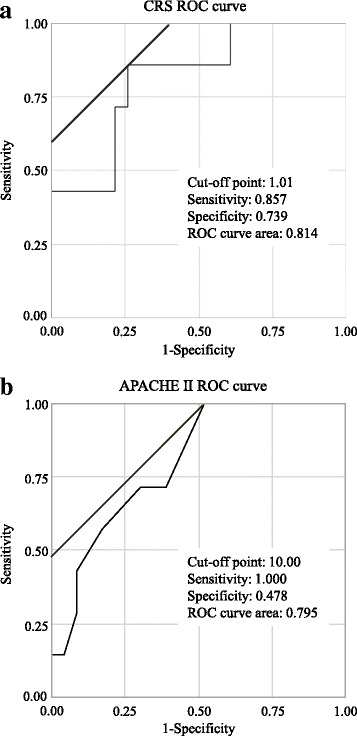
Receiver operating characteristic (ROC) curve of the comprehensive risk score (CRS): (**a**) and acute physiology and chronic health evaluation (APACHE) II: (**b**) for predicting postoperative complications in oral and maxillofacial surgery. Approximate optimal cut-off points for predicting complications: CRS 1.01 (sensitivity: 85.7%, specificity: 73.9%, ROC curve area: 0.814) and APACHE II 10.00 (sensitivity: 100.0%, specificity: 47.8%, ROC curve area: 0.795)

The ROC curve analysis calculated cut-off values of 1.01 for the CRS and 10.00 for APACHE II. The sensitivities and specificities of the CRS and APACHE II were 0.857 and 0.739, and 1.000 and 0.478, respectively. The AUCs of each model for the detection of postoperative complications were as follows: the CRS: 0.814 and APACHE II: 0.795 (Fig. 3).

## Discussion

In the present study, we evaluated the efficacy of the E-PASS and APACHE II scoring systems to predict postoperative complications in the field of oral and maxillofacial surgery. To the best of our knowledge, this is the first study to show the utility of the E-PASS and APACHE II scoring systems for oral surgeons and, thus, may lead to the clinical use of these systems in pre- and postoperative management, surgical decision-making, and informed consent.

Postoperative complications following oral and maxillofacial surgery such as wound infections and swallowing and breathing difficulties have a significant impact on mobility, resulting in prolonged hospital stays and reduced quality of life among patients and, inevitably, increased health care costs [8]. In the present study, seven out of 30 patients were developed postoperative complications; six had pulmonary complications and one had bacteremia. Previous studies have shown that the incidence of postoperative pulmonary complications after surgery ranges between 3 and 48% [9, 10]. Major oral and maxillofacial surgeries with musculocutaneous flap reconstruction involve large skin incisions, thus postoperative pain and tension around the surgical site may contribute to several complications such as atelectasis, hypoventilation, and declined activity. Furthermore, the development of dysphagia in patients who undergo oral and maxillofacial surgery and its detrimental effects on functioning and the quality of life of these patients has been well documented [11]. Oral and maxillofacial lesions are heterogeneous because the anatomic sites of the lesions vary and many factors may influence swallowing process or respiration postoperatively in patients who have undergone oral and maxillofacial surgery. Therefore, surgery in the head and neck area is associated with a high risk of postoperative pulmonary complications [10, 12]. In the present study, those with postoperative respiratory complications had various primary diseases, such as buccal, maxillary, and tongue carcinomas, and carcinoma of the floor of mouth. For the treatment of postoperative respiratory complications, tracheotomy and reintubation were needed in 3 and 1 of the 6 cases, respectively. There were no pulmonary comorbidity and preoperative respiratory function was within normal ranges. Hence, the development of analytical procedures that are sensitive to the factors influencing the relationship between postoperative pulmonary diseases and local states in the head and neck area after surgery are desired.

Previous studies have shown that the most frequent complication in patients who undergo oral and maxillofacial surgery is postoperative infections [13]. Facial reconstruction procedures are immensely complex surgical procedures that are performed to replace tissue defects and restore anatomical structures as a result of various diseases, such as head and neck cancer and oral benign tumors. Therefore, an extensive surgical area is necessary for facial reconstruction and, inevitably, the incidence of postoperative infections is high. In the present study, one patient who underwent maxillary dissection for carcinoma of the maxilla and radical neck dissection developed MRSA pneumonia after 10 days postoperative. Another study revealed that postoperative infections are associated with cardiac disease and diabetes mellitus [14]; similarly, our patient had poor cardiac function (preoperative EF: 29.4%) and diabetes mellitus.

Various scoring systems have been developed to predict postoperative morbidity and mortality. The ideal risk scoring tool has the following attributes: simple; easy to use; reproducible; accurate; reliable; objective; and available to all patients. Hattori and colleagues described the effectiveness of E-PASS scores to predict postoperative complications in colorectal patients [15]. However, only few studies have assessed this system in oral and maxillofacial surgery. We focused on patients undergoing oral and maxillofacial surgery with long operating times who were considered to have more severe surgical stress or a higher risk of postoperative complications than those undergoing minimally invasive surgery.

The CRS was calculated from the PRS (which includes perioperative patient condition factors) and SSS (including surgical condition factors) [2]. In the present study, the CRS was significantly higher in patients with postoperative complications than in those without ones (1.13 vs. 0.90; *p* < 0.01). Previous studies have suggested that patients with CRS > 1 are at a particularly high risk of mortality while those with CRS > 0.5 are at a high risk of morbidity [15]. Furthermore, the CRS, PRS, and SSS correlated with the duration of the hospital stay. These findings suggested that patients with a higher PRS are at a greater risk of perioperative morbidity, particularly in the field of vascular surgery, and the strong correlation between the PRS and outcomes may allow surgeons to predict risks in an individual patient before surgery [15]. In the present study, most patients had CRS > 1.0; only one patient with postoperative complications had a moderately high CRS score (CRS = 0.81). Furthermore, PRS scores were significantly higher in patients with complications. We also used the number of postoperative days until CRP levels decreased to < 1.0 mg/L in order to investigate the improvement outcomes of patients, and the results obtained indicated that a relationship exists between PRS scores and outcomes. Regarding the accuracy of predicting postoperative complications, previous studies demonstrated that E-PASS accurately predicted postoperative mortality in the surgical treatment of hilar cholangiocarcinoma; the AUC to detect in-hospital mortality was 0.842 for E-PASS [16]. Similar results were obtained in our study; we yielded an AUC value for the CRS to predict postoperative complications that was greater than 0.814 and calculated a cut-off value of 1.01, which was consistent with previous findings [16, 17]. Collectively, the results of the present study and previous findings support E-PASS models, particularly the CRS having a high predictive power in the field of oral and maxillofacial surgery. However, the operative type was not a factor associated with postoperative complications in our study; therefore, it remains unclear whether the type of surgery influences the validity of the scores obtained. Nevertheless, some independent factors for postoperative complications, such as operative type, anesthesia type, operative time, anesthesia time, blood loss, and intraoperative circulating volume are expected to contribute to the outcomes of surgery, and further considerations are needed in order to obtain insights into the relationship between intraoperative findings and outcomes.

The APACHE prognostic scoring system is a well-established validated tool for assessing the severity of disease and predicting hospital mortality using data obtained in the ICU admission [5, 18]. APACHE II provides a lot of information on factors influencing the prediction of outcomes, such as age, underlying diseases, and acute physiological conditions, which are crucial for severe morbidity and late mortality; therefore, this scoring system has been frequently used in many ICUs worldwide [6]. Previous studies indicated that the APACHE II scoring system accurately detects hospital mortality by evaluating an AUC of 0.84. Moreover, the best cut-off value in the ROC of this scoring system was 17 [6, 19].

.Therefore, we employed APACE II as an additional indicator to evaluate the clinical status of a patient and predict outcomes after long, highly invasive oral and maxillofacial surgery. As a result, we found that higher APACHE II scores correlated with an increased risk of postoperative complications, and was nearly identical to the E-PASS scoring system. Among our patients, APACHE II scores were significantly higher in patients with complications than in those without ones, a correlation was observed between APACHE II scores and the number of postoperative days until CRP levels decreased to < 1.0 mg/L, and the ACU of APACHE II was 0.795. These results confirmed that APACHE II is a useful parameter that correlates with clinical changes in patients. Nevertheless, previous studies indicated that APACHE II > 17 was associated with a high probability of postoperative morbidity and mortality [6]. In the present study, the median value of APACHE II in patients with complications was 13. A possible reason why our patients had lower APACHE II scores is few of them showed abnormalities in arterial pH, renal functions, or serum electrolytes. Previous studies reported that the predictive accuracy of APACHE system was limited for mortality [19]; accordingly, models that combine additional baseline characteristics or other scoring systems may better assess disease severity, thus, improve the estimated risk of mortality over that with APACHE II alone. Prediction models based on multivariate analyses typically use a logistic regression analysis due to the advantage of its simpler interpretation of the relationship between predictive factors and outcomes [20]. Another study built a prediction model combining APACHE II scores, and found that the new model was more accurate than APACHE II alone for predicting hospital mortality [18]. Difficulties are associated with combining E-PASS and APACHE II scores; however, these scoring models may be useful in the management of patients after surgery; in other words, we recommend the application of E-PASS scores to assess the pre- and intraoperative statuses of patients and APACHE II scores to determine postoperative severity.

The precision and accuracy of surgery are important for all surgeons, and elaborate patient management in the perioperative period is also required in order to prevent postoperative complications. Previous studies have indicated that postoperative prognoses after various surgeries are reflected by the preoperative nutritional conditions of patients [21]. Malnutrition has been identified as an independent risk factor for morbidity and mortality and is associated with a significantly longer hospital stay [22]. Consequently, a good nutritional status is important to avoid postoperative complications in patients undergoing oral and maxillofacial surgery and affects the body’s defense mechanisms in a number of ways. Low levels of serum albumin, nutritional markers such as PNI, and preoperative BMI are major risk factors for adverse postoperative outcomes [17, 23]. These factors have been widely adopted in a large number of facilities, including our own, in order to evaluate the nutritional status of patients. Furthermore, we have adopted more sensitive nutritional markers such as PreAlb, Tf, and RBP, the so-called rapid turnover protein in the past several years; these markers have a half-life in plasma of 2 to 7 days, which is markedly shorter than that of serum albumin [24]. Therefore, PreAlb, Tf, and RBP are more sensitive to changes in the protein-energy status than albumin, and their concentrations closely reflect recent dietary intake rather than the overall nutritional status. In the present study, no significant differences were observed in the nutritional status of patients with/without postoperative complications, and the measured values of nutritional markers mostly remained within normal ranges. This may be attributed to an attempt to intervene in nutritional control in the early phase of the preoperative period with consideration for the importance of nutritional support to prevent postoperative complications. Nevertheless, we cannot exclude the possibility that the incidence of postoperative complications after oral and maxillofacial surgery and, similarly, about 240 million people undergo surgery worldwide, with postoperative complications occasionally reported. In the future, ideal predictors for postoperative complications will be established to assess perioperative conditions from a wider perspective.

A limitation of the present study was that it is a retrospective analysis, which restricts our investigation of data archived during the perioperative period and the number of patients available was small. Consequently, the sample size was insufficient to analyze some intervening variables such as gender, disease, tumor size, and location. Moreover, other factors than that those included in the E-PASS and APACHE II scoring systems could be relevant for predicting postoperative complications and hence, it is important to comprehensively evaluate the risk factor of postoperative complications.

## Conclusions

In summary, we herein analyzed the predictive powers of the E-PASS and APACHE II scoring systems in patients undergoing oral maxillofacial surgery. Our results suggest that their predictive values are promising for oral surgeons. The E-PASS and APACHE II scoring systems will be useful for surgical decision-making, informed consent, and assessing the quality of care in this field. These efforts will improve the quality of surgical performance.

## Abbreviations

APACHE: Acute Physiology, Age, and Chronic Health Evaluation
CRP: C-reactive protein
CRS: Comprehensive risk score
E-PASS: Estimation of Physiologic Ability and Surgical Stress
ICU: Intensive Care Units
PRS: Preoperative risk score
ROC: Receiver operating characteristic
SSS: Surgical stress score
